# Pulmonary Rehabilitation Delivered in Low-Resource Settings for People with Chronic Respiratory Disease: Protocol for a Mixed Methods Process Evaluation of a Three-Arm Randomised Implementation Trial (PuRe)

**DOI:** 10.3390/healthcare14213641

**Published:** 2026-10-27

**Authors:** Purva Abhyankar, Selina Ahmed, Eesha Chawan, Muthu Kamarajan Dharani, Sheron Sir Loon Goh, Norita Hussein, Nur Melissa Abdul Khalil, Wei Leik Ng, Rutuja Patil, Bairave Shunnmugam, Nursyuhada Sukri, Kajal Tonde, GM Monsur Habib, Biswajit Paul, Dhiraj Agarwal, Julia Engkasan, Ee Ming Khoo, Roberto Rabinovich, Vicky Hammersley, Hilary Pinnock

**Affiliations:** 1 Usher Institute, University of Edinburgh, Edinburgh EH16 4UX, UK; 2 Sociology Discipline, Khulna University, Khulna 9208, Bangladesh; 3 Vadu Rural Health Program, Community Health Research Unit, KEM Hospital Research Centre, Pune 411001, India; 4 Rural Unit for Health and Social Affairs (RUHSA), Christian Medical College, Vellore 632004, Indiadrbpaul@cmcvellore.ac.in (B.P.); 5 Department of Clinical Pharmacy and Pharmacy Practice, Faculty of Pharmacy, Universiti Malaya, Kuala Lumpur 50603, Malaysia; sherongoh@um.edu.my; 6 Department of Primary Care Medicine, Faculty of Medicine, Universiti Malaya, Kuala Lumpur 50603, Malaysia; 7 Department of Social and Preventive Medicine, Universiti Malaya, Kuala Lumpur 50603, Malaysia; sbairave@um.edu.my; 8 Community Respiratory Centre, Khulna 9100, Bangladesh; 9 Department of Rehabilitation Medicine, Faculty of Medicine, Universiti Malaya, Kuala Lumpur 50603, Malaysia

**Keywords:** chronic respiratory disease, context, implementation research, low-resource settings, mechanisms, process evaluation, pulmonary rehabilitation

## Abstract

Background: Over half of the 550 million people with chronic respiratory disease (CRD) live in low- and middle-income countries with limited access to pulmonary rehabilitation (PR) that could improve exercise tolerance and quality of life. The PuRe trial tests effectiveness of Home- and Centre-based PR versus usual care in four centres across India, Bangladesh and Malaysia. This protocol outlines the methods used in the process evaluation of the PuRe trial, focused on understanding the context, implementation and mechanisms of PR. Aim:To understand how PR was delivered in the four centres, how the delivery was influenced by the local and national context and the likely mechanisms of impact of PR on trial outcomes. Methods: We use a mixed-methods design. Quantitative data will be collected from researcher and therapist logs and validated questionnaires; qualitative data from interviews with patients, therapists, healthcare professionals, managers, and policymakers. Approximately 80 patients and 20–32 healthcare/stakeholders will be interviewed, guided by data saturation. Implementation: This is assessed via PR reach, fidelity, dose and adaptation. Sustainability will be explored in stakeholder interviews. Mechanisms: These are assessed by patient engagement with PR, patient and therapist experiences and mediators (smoking, physical activity, breathlessness). Context: This is assessed using the Consolidated Framework for Implementation Research exploring how setting, individuals and implementation processes shape delivery and outcomes. Quantitative and qualitative data will be analysed separately and later combined in a mixed-methods synthesis for the purposes of triangulation and complementarity. Discussion: Alongside the trial evidence on effectiveness, an understanding of when, why and how PR works gained through the process evaluation will be valuable for those implementing similar services in other low-resource settings. Trial registration: ISRCTN 65701828.

## 1. Background

More than half of the world’s 550 million people with Chronic Respiratory Diseases (CRDs) live in low- and middle-income countries (LMICs), where mortality and morbidity rates are highest, placing a disproportionate burden on these populations [1,2]. CRDs (e.g., chronic obstructive pulmonary disease (COPD), asthma, interstitial lung disease and post-tuberculosis) are the third leading cause of death worldwide [2]. Socio-environmental determinants of CRDs in LMICs, such as high levels of exposure to indoor and outdoor air pollution, tobacco smoking, rapid urbanization, occupational exposures, low health literacy and socio-economic status, high stigma, and limited healthcare access exacerbate CRD prevalence and hinder effective management [3]. CRDs are associated with reduced quality of life due to breathlessness and fatigue imposing limitations on physical activity. This deconditioning, along with frequent exacerbations requiring medical intervention, can result in anxiety and depression, social isolation, reduced productivity and financial strain [4,5,6].

Pulmonary rehabilitation (PR) is a comprehensive, multi-disciplinary intervention that reverses the deconditioning induced by CRDs, thereby improving exercise capacity, daily functioning and quality of life in people with CRDs [7,8]. PR involves individually tailored exercise training, self-management education, and lifestyle change counselling [9]. However, PR is rarely available or accessible to people in LMICs in part because most of the evidence is disease specific and generated in high-income countries, with uncertain transferability to LMICs where CRDs are often poorly differentiated [10]. Further, PR services may not be deliverable in LMICs and other low-resource settings in the same way as they are delivered in high-income settings due to differences in disease profiles, resources, healthcare configuration, patient and professional awareness, and staff skill mix [11,12]. Practical challenges due to specific clinical, community or home settings in LMICs may require significant adaptations. Before PR can be routinely delivered and scaled up in LMICs and other low-resource settings, more evidence is required not only on whether it can be (cost-)effectively delivered but also on how it can be adapted, optimised and implemented in these settings [13].

The PuRe trial [14] addresses this evidence gap through a three-arm randomised controlled trial (RCT) evaluating whether provision of PR in LMICs improves functional exercise capacity and quality of life among people with CRD compared to usual care (standard care usually available to individuals with CRD attending respective centres). Two modes of PR delivery are tested: PR delivered in a healthcare centre (Centre-PR) or delivered remotely in patients’ homes (Home-PR) over eight weeks with two sessions per week (total sixteen sessions). A detailed description of the PR intervention delivered in both modes is presented in Table 1. The two modes of PR delivery have their unique advantages and disadvantages, particularly within the LMIC context. While Centre-PR may offer the benefits of in-person supervision and group support, distance and cost of travel to a healthcare centre has been reported to be a barrier to accessibility [15]. Home-PR improves accessibility by offering convenient local delivery and potentially better adherence, but unstable connectivity and limited home facilities may prove challenging [16]. The increasing evidence to support comparable effectiveness of telerehabilitation in high-income countries [17] warrants comparison of implementation and outcomes across the two modes in LMICs. The trial is being conducted across four centres in three countries in the Global South: two in India and one each in Bangladesh and Malaysia. Alongside the trial, an independent parallel process evaluation (PuRe-PE) is planned to explore how the intervention worked (or not) and gain useful insights required for its real-world implementation, optimisation and scale-up [18].

This study protocol outlines the methods used in the process evaluation nested within the PuRe trial. The main trial protocol is prospectively registered on the UK’s clinical study registry and is available on the ISRCTN website [14]. The PR intervention has two main components: an exercise component involving individually tailored aerobic and resistance exercises delivered by trained physiotherapists; and an education component that addresses lifestyle changes (physical activity, smoking, biomass, nutrition), self-management (breathlessness, daily activities, medications) and psychological wellbeing (stress, emotions, social support) delivered by trained staff with a mix of skills (e.g., psychologists, occupational therapists). Following Medical Research Council (MRC) guidance [19], the process evaluation will explore three key domains: implementation, mechanisms and context. Specifically, we will focus on understanding how PR is adapted, delivered, and optimised in low-resource settings, why it does or does not work, and what contextual factors affect implementation and outcomes. Given the complexity of delivering PR in a new setting (multiple interacting intervention components; multiple actors including patients, carers, referring clinicians, PR therapists, and service managers; introduction of several new behaviours; long intervention period; need for centre and home PR set-up) and the diversity of surrounding contextual factors (staff skill mix, financial and human resources, local health policies and systems, urban/rural set-up), the process evaluation will provide essential data to support interpretation of the trial outcomes.

**Table 1:** Description of the 8-week PR programme using TIDieR [20].

TIDieR	Description	
Brief names	The two approaches to delivering PR are: (1) Centre-PR and (2) Home-PR.	
What? What tailoring?	We have adapted international standards for delivering exercise components of PR [7,8] in low-resource settings (e.g., 10-metre tracks, steps for endurance; improvised weights, resistance bands for strength). Standardised educational resources (e.g., written information, presentations, video clips) and basic psychological support are locally adapted to ensure cultural and contextual relevance. Behaviour change techniques will optimise sustainability of effect.	
Who provides?	Sessions will be led by PR-trained physiotherapists, with some content (e.g., psychological support, inhaler technique training, smoking cessation) delivered by other professionals according to local skill mix. In-person and online training will be provided to PR staff with ongoing oversight and support from PR experts (RR and GMH).	
How?	All assessments (baseline and follow-up) in all groups will be conducted in the centre. A centre-based session will initiate the prescribed exercise and education programme for participants allocated to Centre- or Home-PR with subsequent sessions delivered according to allocation. A paper-based PuRe pack [21], with tailored exercise prescription and lifestyle and self-management education, will be given to participants. Other video/audio/paper resources about CRD, PR, and exercise demonstrations will support the tailored educational content.	
	Centre-PR sessions last up to two hours and include exercise components and education. In three centres these will be rolling programmes so that participants are recruited continuously and can be allocated to a session within 2 weeks of being randomised. CMC Vellore, India, will recruit participants in cohorts who will be randomised and attend a PR programme as a group.	Home-PR will include the same components as Centre-PR but (after the initial introductory centre-based session), supervision will be delivered remotely. Ideally, we will provide supervision and education via video-links, but availability of technology varies, and we will offer a range of options, e.g., audio calls to supervise exercises and provide motivation and individual education.
Where?	Centre-PR will be in a hospital, community clinic or satellite venue: the key criterion is that the patients attend a group session with at least basic equipment and are supervised in person by a healthcare professional.	Home-PR will normally be undertaken at home: the key criterion is that the patient is by themselves (or with a family member) supervised remotely by a healthcare professional.
When? How much?	Both Centre-PR and Home-PR will provide sessions twice a week for 8 weeks—total of 16 sessions.	
How well?	Guided by international standards [7,8] and informed by local feasibility studies [13,22,23], and stakeholder engagement, we have defined the core components of PR to which fidelity is expected. Practical adaptation to local context is encouraged to enable delivery of the required content (e.g., in Bangladesh single-sex groups are culturally appropriate). Fidelity and adaptation are considered at two levels: Professional fidelity to the manualised PR service. Fidelity will be monitored, and local adaptations recorded. Process data collected will include: therapists’ detailed logs of the exercise and education components delivered to individual patients; qualitative interviews with patients and PR staff. Patient fidelity to the Centre-/Home-PR. We will use the detailed PR service records and explore adherence in qualitative interviews.	

## 2. Aim and Objectives

The process evaluation aims to understand the process of PR delivery in the trial, the local/national context surrounding delivery, the mechanisms of PR’s impact, and future potential for sustainable implementation in routine practice.

The specific objectives are:

1. To assess the implementation of PR in centre- and home-based PR arms within the trial and the potential for future sustained implementation.
2. To explore the mechanisms of impact leading to successful or unsuccessful delivery and outcomes.
3. To examine the influence of context on the implementation, effectiveness and scalability of the PR intervention.

## 3. Trial Setting

The trial will be conducted in the four centres across three countries in the Global South (Bangladesh, India (x2) and Malaysia) who developed PR services within the NIHR Global Health Research Unit on Respiratory Health (RESPIRE) [24] and collaborated to assess feasibility and secure funding for the PuRe trial. Their diverse contexts are detailed in Table 2.

## 4. Overview of Process Evaluation Design

The mixed-methods process evaluation will be guided by the MRC framework for process evaluation of complex interventions [19] and the Consolidated Framework for Implementation Research (CFIR) [25]. Using a convergent mixed-methods design [26], the qualitative and quantitative data will be collected and analysed in parallel throughout the trial period. The MRC framework enables a focus on understanding implementation (what is actually delivered and how delivery is achieved), mechanisms of impact (how the delivered intervention produces change) and context (how the context affects implementation and outcomes). The CFIR provides a structured approach to exploration of implementation determinants across five domains: innovation, inner and outer settings, individual, and implementation processes. A description of how implementation, mechanisms and context will be assessed, guided by the CFIR, within the process evaluation of the PuRe trial is provided below.

### 4.1. Implementation

The term implementation refers to the process or means by which an intervention is “put into use” in a setting. However, the stage of the intervention’s lifecycle determines the types of processes that are investigated under implementation. Implementation during the post-evaluation stage refers to the scaling up or integration of the intervention into routine practice, whereas implementation during the evaluation stage refers to the delivery of the intervention within the trial. In the PuRe process evaluation (PE), we distinguish between and explore both the actual delivery of PR within the trial (trial delivery) and the potential for future sustained implementation and scaling up of PR within the local healthcare systems (future implementation).

#### 4.1.1. Intervention Delivery Within the Trial

The primary aim of process evaluations nested within trials is to assess both the quality (fidelity) and quantity (dose) of what was delivered in practice, the extent to which the intervention reached its intended audiences (reach) and reasons for any adaptations (adaptation). These are operationalised as:

1. Reach. The extent to which the intended target audience comes into contact with the intervention—it will be assessed by collecting data on the response, participation and attrition rates to the trial, and the socio-demographic and clinical characteristics of the participants.
2. Fidelity and dose. We will assess these at two levels. At the patient level, we will assess the extent to which patients engaged with the PR intervention by examining uptake, engagement and completion rates with reasons and explore barriers to and facilitators of adherence. At the professional level, we will assess the recorded quantity of the PR delivered and the observed quality of the delivery process. The quantity and quality of PR delivery will be assessed against the core components of PR specified in a detailed manual (PuRe manual), adapted for LMIC delivery from international PR guidelines [7,8], the PuRe team’s previous feasibility work [13,16,22,23] and local and international expert input (authors RR and MH).
3. Adaptation. We will explore what adaptations were made to the delivery of Centre- and Home-PR and why and whether they represent “innovation”, i.e., changes made to make the intervention fit better with their setting or population; “drift”, i.e., unintentional changes made due to the barriers to full implementation; or “subversion”, i.e., intentional decisions not to adopt aspects of the intervention that conflict with their values or theories of change [27].

#### 4.1.2. Future Implementation

We will explore the perceptions of various stakeholders involving patients, therapists, managers, and policymakers in each of the centres in relation to the likelihood of PR interventions being sustained in their setting beyond the trial, the potential for scale-up beyond the specific settings, and barriers to and facilitators of both sustainability and future scale-up. We will also explore the specific needs of each healthcare setting in relation to patient awareness, professional training and motivation, and organisational support, routines and resources.

### 4.2. Mechanisms of Impact

Mechanisms of impact refer to any processes or changes triggered by the intervention that lead to successful or unsuccessful delivery and outcomes. In evaluations of complex interventions, participants are rarely passive recipients of the intervention; rather, it is the people involved in the interventions who make them work or not. People’s active interactions with the intervention, influenced by the specific context in which they operate, represent the mechanisms of change. In PuRe-PE we will seek to understand how participants (staff and patients) engaged with, perceived and experienced the PR intervention in the following ways:

1. Potential mediating variables such as change in smoking status, physical activity, anxiety and depression, and breathlessness, which may indicate the extent to which patients engaged with and acted on the PR content.
2. Patients’ experiences of receiving both Centre- and Home-PR, reasons for (non) adherence, barriers to and facilitators of engagement, and views on the effectiveness of PR, use of the educational PuRe pack resource, unanticipated and unintended consequences of PR, and costs to individuals and families.
3. PR therapists’ experiences of delivering both Centre- and Home-PR, barriers and facilitators, and unintended and unanticipated consequences.

### 4.3. Context of Implementation

Context includes anything external to the intervention which influences implementation, participants’ response to the intervention and if/how that impedes or strengthens intervention effects. The Consolidated Framework for Implementation Research (CFIR) proposes five domains to guide the assessment of context: innovation, outer setting, inner setting, individual and implementation process [25]. We will use the CFIR to explore how the contextual factors across these five domains influenced implementation, mechanisms and outcomes (document research and stakeholder interviews).

## 5. Data Collection

The key domains of process evaluation (implementation, mechanisms and context) will be assessed through triangulation and synthesis of data collected using both quantitative and qualitative methods, from a range of sources and at various time points throughout the trial. The qualitative and quantitative data collection will proceed in parallel, with the two forms of data analysed separately and merged at the end of the study period. The data collection plans presented below are grouped into quantitative and qualitative methods (see Table 3 for a summary of data collection activities).

### 5.1. Quantitative Data Collection

Response, participation and attrition rates: The following data will be collected from the trial recruitment logs and REDCap [28] database throughout the trial period.

- Number of patients who were screened for eligibility by the study team and source of their referral (self, clinician, registry).
- Number of patients identified as eligible and ineligible (and reasons for ineligibility).
- Number of patients consenting to the trial (in total and by study arm).
- Number of patients declining to take part in the trial and their reasons (in total and by study arm).
- Number of participants who withdraw from the trial and reasons for withdrawal (trial attrition).
- Number of patients offered information about the trial by clinician or researcher or through outreach activities. This will be an estimated figure as this information will not always be recorded formally (e.g., by a clinician in a consultation or field visits by teams).

Participant characteristics: Data on consenting patients’ demographic and clinical characteristics will be available from the baseline data collected from study participants and entered in REDCap. The following data will enable assessment of equity, diversity and inclusion within the trial participants compared to the target population [29]: age, gender, geographical distance from the centre, diagnosis, severity of CRD, socio-economic status, educational status, smoking status, and co-morbidities. A table of characteristics of the people we expect to recruit is available in the trial protocol [14].

Patient fidelity to Centre-/Home-PR and dose of intervention: Fidelity at the patient level is defined as the extent to which patients engage in Centre- or Home-PR as prescribed by their therapist. Patient attendance at PR sessions will be recorded by PR therapists in paper-based PR-logs and entered by researchers on an Excel database. From these records, we will collect data on uptake, engagement and completion as defined in Uzzaman et al. [16]:

- PR uptake—the number of patients who attended the initial baseline assessment and at least one PR session.
- PR engagement—the proportion of PR sessions attended by patients and reasons for non-attendance. This reflects the dose of intervention. Attending at least 70% of the sessions is considered good engagement [30]. Although most studies support a significant relationship between increased frequency of exercise training and greater improvements in exercise outcomes [31], there is no agreed threshold for the number of sessions that can be regarded as adequate. We will therefore adopt the 70% level used by the UK National Asthma and Chronic Obstructive Pulmonary Disease Audit Programme [30].
- PR completion—the number of patients who attended the PR discharge assessment and are regarded as having “completed” the PR programme (regardless of the proportion of sessions attended), i.e., attended at least one session and the post-PR assessment.

Professional fidelity to the manualised PR service: Both Centre- and Home-PR provide twice-weekly sessions for eight weeks and include two components: exercise (aerobic and resistance) and education (including behaviour change). While the PR therapists are trained in delivering the Centre-/Home-PR intervention in a standardised manner, they are expected to prescribe and deliver a personalised exercise and education programme to the participants tailored to the individual’s disease and severity, supported by a personalised resource pack (PuRe pack) [21]. While tailoring and adaptation are expected, fidelity to the core components of PR specified in the PR guidelines can be monitored [7]. We will monitor and record what was delivered in practice, how well and how much, what adaptations were made and their reasons.

We will collect the following data from the therapists’ logs:

- Delivery of core components that are essential to any PR programme to assess fidelity.
- Type, duration and increasing load of exercise components in both Centre- and Home-PR and mode of supervision (group/individual; in person, video/audio calls).
- Number, duration and content of education components delivered to individual patients in both Centre- and Home-PR, and mode of delivery (group/individual; in person, video/audio calls).

We will use non-participant observation of a Centre- and a Home-PR session during the quality assurance site visit by HP and VH after recruitment of the first 30 patients.

- A checklist will be developed to record the quality of PR delivery.

Potential mediating factors: The PuRe trial will assess several intermediate outcomes which may mediate the effect of PR on clinical outcomes such as exercise capacity and quality of life, thereby acting as mechanisms of impact. Potential mediators, including smoking status, physical activity, and breathlessness, will be assessed as secondary outcomes in the trial using validated questionnaires (see PuRe trial protocol [14]).

### 5.2. Qualitative Data Collection

Qualitative data collection includes semi-structured interviews with participating patients, PR staff, healthcare professionals providing care for CRD, service managers and other relevant stakeholders (see Supplementary Materials for all topic guides). All interviews will be conducted by an independent local team of qualitative researchers, audio-recorded and transcribed verbatim in participants’ preferred language. Analysis will be in the local language to preserve the meaning with quotes and sections of transcripts translated to English by the research team for discussion with the multinational team and inclusion in reports and publications.

#### 5.2.1. Semi-Structured Interviews with Patient Participants and Caregivers

##### Sampling and Recruitment

The eligibility criteria include all patient participants who have consented to take part in the PuRe trial and are between 3 and 6 months post randomisation. The sample size will be guided by data saturation (i.e., ending data collection when no new themes are identified) and information power (ending data collection when sufficiently rich information is provided by the sample); however, in each centre, we plan to recruit a maximum of 20 participants from the two study arms—Centre-PR (n ≈ 10), Home-PR (n ≈ 10). Sampling size is informed by previous qualitative implementation studies and is expected to achieve thematic saturation within and across centres. Maximum variation sampling will be used to identify and invite participants based on: age, gender, socio-economic status, geographical distance from the centre, specific CRD diagnosis, multimorbidity, time since randomisation and completion/non-completion of the PR program. If additional factors emerge as important in one centre (e.g., presence/support of a caregiver) we will include this in our sampling for other centres. Data saturation is considered reached when no new themes are identified across three consecutive interviews.

A trained local qualitative researcher will identify potential participants from each study arm from trial recruitment logs using the above sampling frame. Consent to approach potential interviewees is included in the trial recruitment consent form. Initial approach with potential participants will be by email, text, phone, letter or in-person (according to recorded preference) in which they will be provided with a written information sheet giving details of what is involved in the qualitative study. Participants will have at least 24 h after receiving an information sheet to decide whether or not to take part. To ensure understanding, the researcher will explain the qualitative study and answer any questions before taking informed consent.

##### Procedure

Interviews will be conducted at a mutually convenient time and venue (home, local clinic or community space). Although face-to-face interviews are preferred with participants, telephone or video interviews may be arranged if that enables participation (e.g., to fit around work or domestic commitments). On the day of the interview, the researcher will ensure the participant has understood this study, answer any questions and obtain written informed consent. Participants being interviewed remotely will be asked to send the signed consent form to the researcher by email or post. If written consent cannot be obtained prior to the interview day, verbal consent will be obtained.

Ideally interviews will be with the participant alone, but we recognise that caregivers may have a significant role in supporting people with more severe CRD. In some interviews the caregiver may be present de facto. In all situations the preference of the participant will be the priority, and arrangements will be recorded in the interviewer’s field notes as context for the interview. Interviews are expected to last between 30 and 60 min.

##### Topic Guide

The topic guide for participant interviews will be designed to collect data on their experiences of engaging in PR and views on its impact (i.e., potential mechanisms of action). It will be informed by the CFIR framework and earlier qualitative work in our feasibility studies [22,23,32,33], which showed that people with CRD were often not aware of the diagnosis and defined their condition by the symptom (typically breathlessness) as opposed to a recognised disease. This could lead to poor acceptance of an intervention such as PR which promotes exercise that in the short term makes breathlessness worse. Participants also described practical difficulties of attending PR sessions at a centre but conversely fear of triggering an attack of breathlessness while undertaking exercise at home. Topics covered will include understanding and experience of living with CRD, views and experiences of engaging with the PR course, reasons for adherence/non-adherence to the PR programme, barriers to and facilitators of participating in PR, patient-led adaptations to PR, and suggestions for future improvements.

#### 5.2.2. Semi-Structured Interviews with PR Staff

##### Sample and Recruitment

Staff in each centre who were involved in the delivery of PR (Centre-PR or Home-PR) in any way will be eligible and invited to take part in PR staff interviews. The sample will mainly include PR therapists, but also other staff (e.g., psychologists, nutritionists) according to the skill mix in the local centres. The number of PR staff in each centre is expected to be between 5 and 7 (between 20 and 28 overall).

Staff will be invited to take part in individual interviews by the qualitative researcher by email or letter at the beginning of the trial. They will be provided with a written information sheet and written consent will be obtained prior to interview.

##### Procedure

PR staff will be interviewed twice during the trial period—once at the end of about 3 months, when the first 30 patients have been recruited and again at the end of the intervention period. The interviews are expected to last between 20 and 45 min, will be undertaken by telephone, video link or face to face in a mutually convenient venue (office, local clinic).

##### Topic Guide

The topic guide for PR staff interviews will be designed to collect data on staff’s experiences and views on delivering PR and its potential impact (i.e., potential mechanisms of action), views on the training, resources, support and wider organisational/health system factors impacting on delivery (i.e., context) and views on sustained implementation (i.e., future implementation). Topics covered will be informed by the CFIR framework and include understanding of CRDs and the role of PR, practicalities of and barriers to and facilitators of delivering Centre- or Home-PR, experience of delivering all PR components and observed benefits, adaptations made and reasons, views on PR training received, and suggestions for improvement and considerations for sustainability.

#### 5.2.3. Semi-Structured Interviews with Health Professionals, Service Managers and Other Stakeholders

##### Sample and Recruitment

In each centre, the sample will include health professionals who care for people with CRD (hospital- and community-based, specialists and primary care), healthcare/hospital managers and other stakeholders (as identified within the stakeholder mapping and activities). We aim to interview a total of 5 to 8 staff across these roles (maximum 20 to 32 overall).

Staff will be invited to take part in individual interviews by the qualitative researcher by email or letter at the beginning of the trial. They will be provided with a written information sheet and written consent will be obtained prior to interview.

##### Procedure

The above staff will be interviewed once at the end of the intervention period. The interviews are expected to last between 20 and 45 min, will be undertaken by telephone, video link or face to face in a mutually convenient venue (office, local clinic).

##### Topic Guide

The topic guide for healthcare professionals, service managers and other stakeholders will be designed to collect data on the context and future implementation of PR, informed by the five domains of the CFIR framework. It also builds on earlier qualitative work in our feasibility studies [15,22,23,32,33,34,35], which suggested that non-respiratory clinicians had limited knowledge of PR, sometimes confusing it with chest physiotherapy. Multiple barriers were practical (lack of facilities/expertise) but also organisational (lack of referral pathways, no reimbursement). Topics addressed cover awareness and understanding of CRD, usual care of patients, awareness and understanding of the role of PR, views on its effectiveness and feasibility, barriers to and facilitators of referring patients with CRD to PR and to sustained implementation of PR in the local health system and future scale-up. Once initial perceptions have been discussed, information about PR will be provided for those stakeholders unfamiliar with PR. To mitigate the risk of priming or social desirability bias, the PR information will be framed using neutral language giving a factual overview of PR, avoiding a persuasive tone. Subsequent questions will focus on the applicability of PR within the participant’s specific local organizational context (e.g., staffing, infrastructure, referral pathways), leveraging their professional expertise to identify real-world barriers and facilitators rather than soliciting general attitudes.

## 6. Data Analysis and Synthesis

We will use established mixed-methods approaches [26,36], initially analysing the quantitative and qualitative data separately, then undertaking a mixed-methods synthesis.

The main trial outcomes will be entered into a REDCap database [28] held centrally and securely in the Edinburgh Clinical Trials Unit. The main trial analysis will be conducted by the trial statisticians and is detailed in the statistical analysis plan that will be made available on the trial registration [14]. This includes a complier average causal effect (CACE) analysis which aims to estimate the causal effect of the intervention among participants who adhere to the intervention as intended. Post hoc mediation analyses may be conducted to explore whether changes in secondary outcomes (breathlessness, physical activity, smoking status, and anxiety/depression) mediate the intervention’s effect on the primary outcomes. The anonymised data on PR delivery collected from therapist logs will be entered into an Excel spreadsheet by local researchers with at least 20% of data checked by another researcher for accuracy and consistency. These will be transferred from the countries to the University of Edinburgh in the UK via a secure, password-protected online platform for storage and analysis. These data will be analysed descriptively to report the extent of PR’s reach and delivery, the nature of PR components delivered, and the extent of patient uptake, engagement and completion. Inferential statistics will be used to examine variations in these process measures by study arms, centres, participant characteristics, asthma severity, and co-morbidities.

Anonymised qualitative data will be stored in the local centres on their secure systems for five years, only accessible to local process evaluation teams. Working closely with the process evaluation lead to ensure consistency throughout the analytical process, qualitative data will be analysed at the centre level by local research teams using thematic and framework analysis. Data from each stakeholder group (patients, therapists and CRD professionals/service managers/policymakers) will be analysed separately, guided by the five domains of the CFIR framework (innovation, inner and outer settings, individual and implementation process) using a combination of inductive and deductive thematic analyses and locally available data analysis software (e.g., NVivo or Atlas.ti). A framework approach, using MS Excel/Word, will then be used to plot the key themes from each group across the CFIR and other inductively developed domains. This will enable us to compare and contrast the accounts of different stakeholder groups, identify convergent and discordant views and experiences, and provide a comprehensive story of implementation specific to each centre, enhancing the credibility and transferability of the data. Analytical consistency and dependability will be maintained across the centres through regular team meetings, standardisation of analytical process, use of audit trails, bespoke training of researchers and timely troubleshooting. The qualitative findings from the centres will be shared in the form of a report with the process evaluation lead, who will then undertake a qualitative meta-synthesis of the themes emerging from the four centres to derive high-level themes applicable more widely in similar settings. We will use a deductive approach informed by the CFIR framework and a trial-specific logic model before combining with an inductive approach in which we will build and test emergent theories from the data as they appear. Cross-centre comparisons will focus on identifying specific mechanisms of impact that lead to positive or negative outcomes and the contextual factors that enable or disable those mechanisms.

The cultural and linguistic diversity across the four centres poses significant operational challenges. To preserve the nuance and cultural context of participant experiences, interviews will be conducted and initially analysed in the local languages by local researchers rather than being immediately translated into English. While this approach mitigates the risk of translation bias during the coding phase, it introduces complexities in synthesising findings across sites and ensuring consistent interpretation. The local bilingual teams will translate coding frames, analytical summaries and illustrative quotes into English for central review. Regular calibration meetings between the site leads and the central process evaluation team will be utilized to negotiate meanings and ensure that cultural specificities are accurately represented in the final synthesis.

Once qualitative and quantitative data have been analysed and synthesised across centres separately, the findings will be integrated through merging of the two types of data at the interpretation and reporting level [26]. We envisage integrating the findings through a narrative weaving approach where qualitative and quantitative findings will be reported together on a theme-by-theme or concept-by-concept basis. Integration will be used for the purposes of triangulation (i.e., quantitative and qualitative findings compared for consistency of findings) and complementarity whereby the qualitative findings will be used to expand, explain or qualify the quantitative results. As the two forms of data investigate different but complementary aspects of the same phenomenon of interest (e.g., quantitative data explore the nature and extent of PR delivery and completion while qualitative data explore experiences, adaptations, barriers and facilitators), they will be integrated to expand the insights offered by one another. For instance, quantitative data may indicate lower PR completion rates for older patients in Home-PR, but qualitative data will help explain this pattern by offering insights into barriers to engagement. Depending on the nature of the emergent findings, visual integration methods, such as joint displays or side-by-side comparisons, will be utilised to enhance the clarity of the triangulation and complementarity processes during the reporting phase.

### Logic Model Development

**Figure 1:**
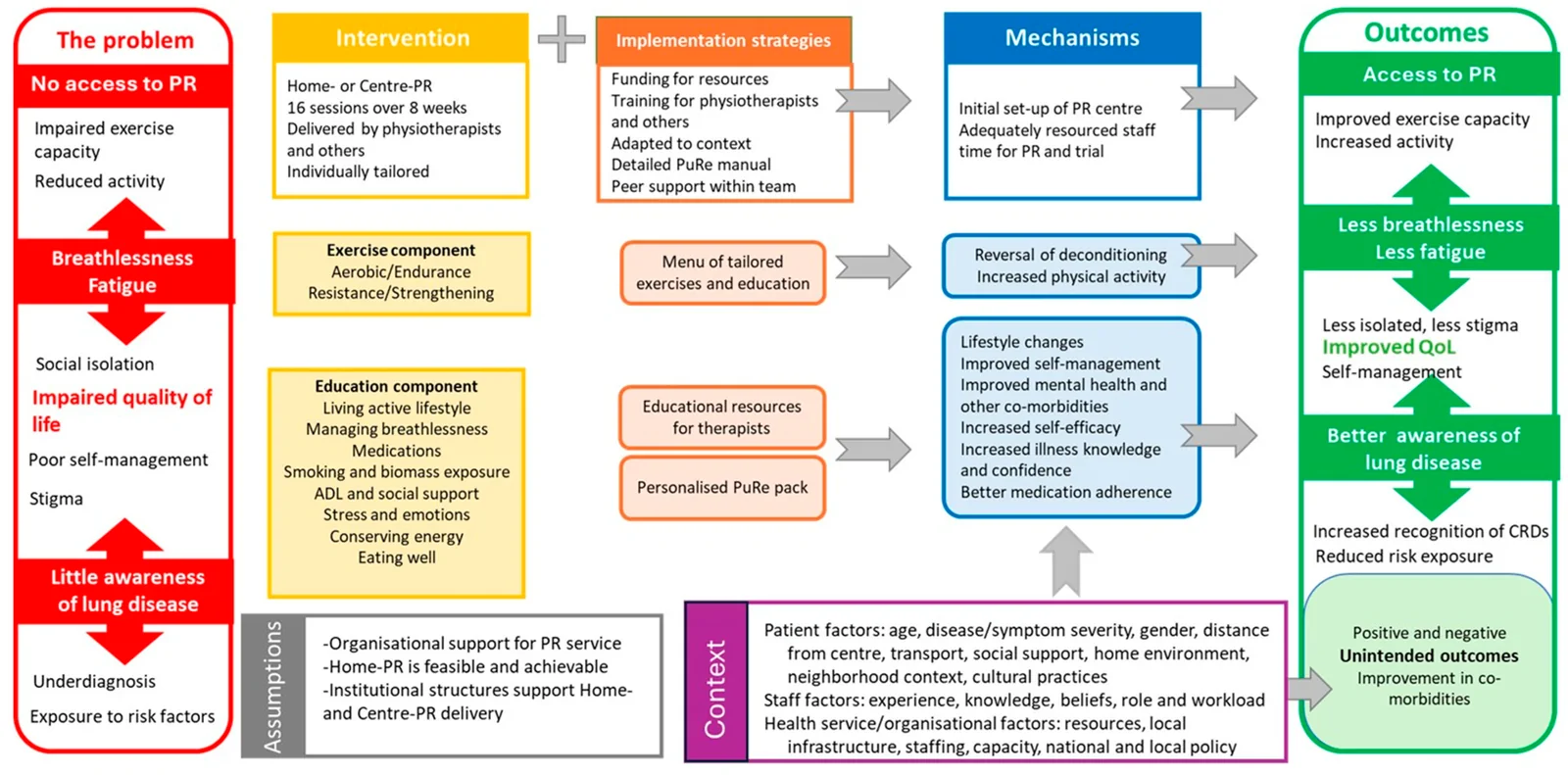
Logic model for Centre- and Home-PR. Note: This logic model will be used as a starting point and revised and refined using the findings from the process evaluation.

The MRC guidance on process evaluation [19] recommends developing a logic model to clarify the assumptions about how the intervention will produce the intended effects, using this “intervention theory” to inform data collection, and refining the theory in light of the findings. A logic model is a diagrammatic representation of the relationships between an intervention’s resources, activities and intended outcomes [37]. We will develop a logic model describing how the PR intervention is expected to work based on the trial hypotheses and the existing literature and refine it iteratively throughout the process evaluation in response to emerging data, both qualitative and quantitative. We will also investigate whether the “theory of change” is different across the four centres, given the diversity of settings and contexts and likely differences in implementation processes. Figure 1 presents an initial logic model illustrating how the PR intervention and the implementation strategies used to support its delivery may activate a series of mechanisms of action that lead to both anticipated and unanticipated outcomes. It outlines potential contextual factors, at the patient, staff and service level, which may influence the implementation process, mechanisms of action and outcomes. For instance, patients’ engagement in PR may enable them to make lifestyle changes (e.g., quit smoking, increase physical activity), boost their confidence in performing daily activities, and improve their self-management, anxiety and low mood, which may in turn improve their quality of life and have a beneficial effect on their co-morbidities such as diabetes/hypertension control. However, the level of social support patients have may influence their ability to engage in PR sessions at the centre (e.g., support with travel) or at home (support with mobile phone use), ultimately influencing the benefits they can acquire through PR.

## 7. Community Engagement and Involvement

Community engagement and involvement [38] has been at the core of the PuRe trial from its inception [32,34], including study design and trial oversight. This is described in the main trial protocol [14].

## 8. Discussion

A key strength of this process evaluation is that the researchers collecting process evaluation data are independent of those delivering the trial and the PR intervention, reducing the risk of bias. Through regular group meetings with the process evaluation lead, the researchers are enabled to adopt a balanced and critical lens and maintain the confidentiality of the process data when communicating with trial teams. Any formative feedback relevant to PR delivery/organisation emerging from patient or therapist interviews is communicated to relevant teams centrally, constructively and anonymously. An important limitation is the lack of interviews with patients from the usual care arm, which would have allowed comparison with views from those receiving usual care.

The mixed methods process evaluation conducted alongside the effectiveness trial will help explain under what conditions and in what ways the implementation of PR in low-resource settings worked or not. In particular, it will help clarify the processes of PR intervention, and shed light on how home-based PR and centre-based PR were implemented, adapted and optimised for the needs of the local settings. It will also describe the extent to which the core components of the intervention were delivered, the extent to which patients engaged with PR components and found them beneficial and acceptable, and the barriers to and facilitators of implementation and how they were overcome. Finally it will inform the likelihood of PR service being sustained and scaled up beyond the trial period. Together with the evidence on effectiveness, this knowledge of acceptability, feasibility, adaptation and sustainability will bring practical and useful information for healthcare teams in similar settings planning to implement PR for people with CRDs.

## 9. Conclusions

This process evaluation will offer critical insights into the adaptation and implementation of centre- and home-based pulmonary rehabilitation for people with CRDs in low-resource settings. Combined with effectiveness data from the main trial, the findings will offer actionable insights into the sustainability and scalability of PR services beyond the trial period, equipping healthcare teams in similar contexts with the necessary knowledge to implement PR effectively.

## 10. Trial Status

Recruitment for the PuRe trial began on 29 May 2025 and is scheduled to complete in August 2026. Recruitment for the process evaluation began in August 2025 and is scheduled to complete in February 2027.

## Figures and Tables

**Table 2:** Features of the four participating centres.

Centre (World Bank Status)	Khulna, Bangladesh (LMIC)	CMC, Vellore, India (LMIC)	KEM, Pune, India (LMIC)	Kuala Lumpur, Malaysia (UMIC)
Features of the four centres				
Setting	Community-based respiratory outpatient care with PR facility	Rural Unit for Health and Social Affairs In/outpatient facility	Rural Health Program (Vadu). In/outpatient facility.	Tertiary hospital, urban setting. In/outpatient facility.
Staff	Primary care physician, medical assistant, physiotherapists	Physicians, physiotherapists, psychologist, nutritionists, occupational therapist	Respiratory physicians, physiotherapists, psychologist	Rehabilitation physicians, physiotherapists, occupational therapist, dietitian

**Table 3:** Summary of data collection during PuRe process evaluation.

Domain	Source	Timescale
**Implementation (delivery within trial and future implementation)**		
Reach (proportion of eligible patients exposed to PR)		
No. of patients screened, identified as eligible/ineligible, consented/declined, withdrew from trial	Trial recruitment logs	Throughout recruitment phase
Participant characteristics	Trial baseline data	Baseline
Fidelity (adherence to PR core components)		
Fidelity (patient)		
PR Uptake, Engagement, Completion	Therapist logs	Throughout trial
Fidelity (Therapist)		
Quantity of PR delivered (no. and duration of exercise and education components delivered)	Therapist logs	Throughout trial
Quality of PR delivered (observation of PR sessions)	PR delivery checklist	Pilot phase of the trial
Adaptation (modifications to intervention)		
Adaptations made to PR delivery	Interviews (therapists)	
Sustainability		
Likelihood of PR to be sustained in the setting and potential for scale-up	Interviews (therapists, HCPs, managers, other stakeholders)	End of intervention period
**Mechanisms of impact (processes or changes triggered by the intervention that lead to successful or unsuccessful delivery and outcomes)**		
Mediating variables (change in smoking, activity, breathlessness)	Questionnaires	12 w and 26 w post randomisation
Patient experience of receiving PR	Interviews (patient)	3 to 6 months post randomisation
Therapist experience of delivering PR	Interviews (therapist)	End of 3-month pilot + End of intervention period
**Context (Anything external to the intervention which influences implementation, mechanisms and impedes or strengthens intervention effects)**		
Five domains of CFIR (characteristics of the innovation, outer setting, inner setting, individual and implementation process)	Interviews (therapists, CRD-healthcare professionals, service managers and others)	End of intervention period

## Data Availability

No new data were created or analysed in this study. Data sharing is not applicable to this article.

## Supplementary Materials

The following supporting information can be downloaded at https://www.mdpi.com/article/10.3390/healthcare14213641/s1: Table S1: Patient interviews topic guide; Table S2: PR therapist interviews topic guide; Table S3: Other stakeholder interviews topic guide: healthcare professionals involved in CRD care; Table S4: Other stakeholder interviews topic guide: managers, policymakers, community leaders.

## Abbreviations

CFIR	Consolidated Framework for Implementation Research
CRD	Chronic Respiratory Disease
COPD	Chronic Obstructive Pulmonary Disease
LMICs	Low- and Middle-Income Countries
MRC	Medical Research Council
PR	Pulmonary Rehabilitation (as in Centre-PR and Home-PR)
RCT	Randomised Controlled Trial
REDCap	Research Electronic Data Capture
