# Supplementary material for: Pulmonary Rehabilitation Delivered in Low-Resource Settings for People with Chronic Respiratory Disease: Protocol for a Mixed Methods Process Evaluation of a Three-Arm Randomised Implementation Trial (PuRe)
Source: Healthcare (Basel). 2026 Oct 27;14(21):3641. doi: 10.3390/healthcare14213641 (PMC13762836; doi:10.3390/healthcare14213641)
Supplement: Supplementary file 1 [file healthcare-14-03641-s001.zip › healthcare-4600700-supplementary.pdf]

Supplementary material - Topics guides

Table S1: Patient interviews topic guide

Main theme	Sub-themes and prompts
Living with CRD	
Awareness and understanding of CRD	Diagnosis?<br>• What do you call the condition?<br>• Has anyone explained the condition to you (before and/or during pulmonary rehabilitation)?<br>• What helped (or worried you about the explanation)? Why?<br>• What do your family, friends, colleagues know about your condition? (is this helpful/a problem? Why
Symptoms and impact of living with CRD	Which symptom(s) trouble you most? (breathlessness, cough, sputum, fatigue?<br>• How do these symptoms affect your day-to-day life? (home, family, hobbies, leisure, work?)<br>Tell me about what it is like to experience of living with chronic symptoms (especially etc)
Personal goals	What did you hope that pulmonary rehabilitation would help you achieve?<br>• What did you achieve after rehabilitation?<br>• What was not achieved?
Views on the PR course	Centre- or Home-PR?
Exercise training	Impact on symptoms<br>Impact on activities (examples?)<br>Confidence (or not) in undertaking exercise
Education programme	Consider: Inhaler technique, Breathing techniques, Coping strategies, Self-management, Nutritional advice, Smoking and biomass, Psychosocial impact
Service considerations	
Practicalities	How did you find the practicalities of participating the pulmonary rehabilitation?<br>• Convenience? Making time?<br>• Advantages (or not) of being part of a group?<br>• Family support (or not)?<br>• Home exercise/practice<br>• Remote communication (how/ did it work/any problems?)
Implementation and sustainability?	How could the service be improved?<br>Suggestions for future improvements?
Summary	
Overall benefits	Overall benefits (or not) to health and well-being
Completion	Reasons (if any) for persisting or withdrawing from the PR programme
Goals	Achieve original goals? Examples?<br>If not what prevented achieving goals
Any other comments?	

Table S2: PR therapist interviews topic guide

Main theme	Sub-themes and prompts
Delivering the PR course	
Practicalities of delivery	• Ease or difficulty in setting up the course for center-based and home based<br>○ On a practical level how well did the Centre-PR and/or Home-PR work? Barriers and facilitators in delivering PR<br>○ Convenience? Making time?<br>○ Advantages (or not) of group delivery<br>○ Supervision of Home exercise/practice<br>○ Remote communication (any problems?)<br>○ Education delivery (practical issues)<br>• Any safety concerns during PR delivery? Home and Center?
Experience of PR delivery	• Confidence and skills in delivering exercises training, education and behaviour change support<br>• Experience of tailoring the exercise and education<br>• Change in confidence and skills with practice<br>• How PuRe pack was used<br>• Management of adverse events<br>• Factors (or lack of) that helped with effective delivery (e.g. support, supervision, time, resources)
Adaptations	• Any adaptations made?<br>• Reasons, effect on delivery
Barriers and enablers to patient adherence	• Factors making it easier or harder for patients to engage with PR<br>• Reasons for persisting or withdrawing from PR course<br>• Any differences in engagement by gender, age, occupational or educational status<br>• Any suggestions from patients for future improvements?
Views on the PR course	
Benefit of PR	• Views on benefit/usefulness of PR in<br>○ Improving exercise capacity/fitness<br>○ Increasing self-confidence<br>○ Improving self-management<br>○ Improving other conditions/aspects of life<br>• Any other consequences of PR – positive or negative?<br>• Views on benefits of the two modes of delivery (home and center)?
Patient feedback on PR	• Any patient feedback on usefulness/benefit of exercise training<br>• Any patient feedback on usefulness/benefit of education<br>• Usefulness of PuRe pack<br>• Any concerns from patients?
PR training & resources	
Views on PR training	• Need for training<br>• Usefulness and adequacy of training – exercise and education delivery

	• Preparedness and confidence as a result of training<br>• Which mode of training was helpful? In person or online?<br>• Any unmet training needs?
Views on PR resources	• PuRe pack<br>• PuRe manual & slides<br>• Educational resources for use with patients
Future Implementation and sustainability	
Future Implementation	• What would you change about how the PR was delivered? (exercise as well as education components), Why<br>• What would you need for improved delivery in future? (resources, time, support, other factors)
Sustainability	• How could the PR service be sustained in your setting?<br>• How could a PR service be rolled out in <country>?
Summary	
Overall benefits	Perception of overall benefits (or not);<br>• to patients health and well-being<br>• to healthcare service in <country>
Any other comments?	

Table S3: Other stakeholder interviews topic guide: healthcare professionals involved in CRD care

Main theme	Sub-themes and prompts
CRD, and the role of PR	Note: Modified according to the professional group
Awareness and understanding of CRD	What conditions do you regularly treat, diagnose?<br>• How common are respiratory issues among your patients?<br>• How do you confirm the diagnosis?<br>• Do you, or one of your staff, explain the condition to the patient (before and/or during pulmonary rehabilitation)?<br>• What are the barriers? (Time, resources, costs?)<br>- Barriers to confirmation of diagnosis<br>- Barriers to explanation of the condition to patient<br>• How are CRDs/Respiratory issues usually treated in your setting?
Awareness of PR	Were you aware of PR prior to this trial? If no, give information about PR.<br>• To what extent is PR needed in your setting?<br>• What evidence were you aware of PR being effective for patients with CRDs?<br>The research evidence for PR is considered to be very strong, but do you think there is more evidence that clinicians from <country> need before it can be made to work in practice?
What do you understand as the role of pulmonary rehabilitation?	When do you suggest PR for your patients (or not)?<br>What do you hope that pulmonary rehabilitation will achieve for your patients?<br>If you do not refer for PR, what are the barriers?

[Information available for those less familiar]	• Unfamiliar with contra/indications?<br>• Uncertain exactly what happens in PR?<br>• Not convinced of its effectiveness<br>• No local service/no good quality service,<br>• No referral pathway?<br>• Safety concerns?<br>• Patient/carer refusal, low need
Views on the PR course	Centre- or Home-PR?
Have any of your patients attended PR (PuRe trial)?	Have you had any feedback about the benefit (or not) or PR?
Exercise training	Have you had any patients feedback about the exercise programme?<br>Impact on symptoms/activities (examples?)<br>Any unintended consequences (positive or negative)?<br>Any concerns?
Education programme	Any patient feedback about the education component?<br>Active lifestyle, managing breathlessness, medications, conserving energy, activities of daily life, nutritional advice, smoking and biomass, stress and emotions
Service considerations	
Practicalities	On a practical level how well did the Centre-PR and/or Home-PR work (or how do you think it would work if you have had no feedback). What advantages do you see in Centre- vs Home PR<br>• Convenience? Making time?<br>• Advantages (or not) of being part of a group?<br>• Family support (or not)?<br>• Home exercise/practice<br>• Remote communication (any problems?)
Barriers and enablers	What factors make it easier/harder for patients to engage with PR<br>Reasons (if any) for persisting or withdrawing from the PR programme<br>Suggestions for future improvements?
Implementation and sustainability?	How could a sustainable PR service be implemented and rolled out in <country>?<br>How could a sustainable PR service be implemented and rolled out in <country>?<br>Role of –<br>Physical & technological infrastructure (space, equipment, tele-communication systems)<br>Work infrastructure (staffing levels, roles and responsibilities of staff, working relationships, team work)<br>Incentives supporting implementation<br>Availability of resources/funding<br>Buy-in and Support (from decision/policy-makers, managers, opinion leaders)<br>Motivation (commitment to improve care/outcomes for CRDs)
Summary	

Overall benefits	Perception of overall benefits (or not);<br>• to patients health and well-being<br>• to healthcare service in <country>
Any other comments?	

Table S4: Other stakeholder interviews topic guide: managers, policymakers, community leaders

Main theme	Sub-themes and prompts
CRD, and the role of PR	Note: Modified according to the professional group
Awareness and understanding of CRD	• How much of a problem is CRD in <country>?<br>• Any particular CRDs that you are aware of?<br>• What do you think about the treatment/care for CRDs in <country>
Views on the PR course	Centre- or Home-PR?
What are you views on PR (PuRe trial)?	Have you heard of PR? What do you understand by PR?<br>What do you think/know about whether PR is effective or not?<br>How much of a priority is PR as a treatment strategy in <country>, your community<br>Have you heard about the PuRe trial?<br>Have you had any feedback about the benefit (or not) or PR?<br>• Are you aware of exercise in PR? Examples of impact on symptoms/activities (?)<br>• Are you aware of educational programme in PR? (Active lifestyle, managing breathlessness, medications, conserving energy, activities of daily life, nutritional advice, smoking and biomass, stress and emotions)
Service considerations	
Practicalities	On a practical level the PuRe trial offered Centre-PR and Home-PR work What do you see as the advantages of these options?<br>• Convenience? Making time?<br>• Advantages (or not) of being part of a group?<br>• Family support (or not)?<br>• Home exercise/practice<br>• Remote communication (any problems?)
Barriers and enablers	What factors make it easier/harder for patients to engage with PR<br>Reasons (if any) for persisting or withdrawing from the PR programme<br>Suggestions for future improvements?
Implementation	What could make it easier or harder for services to deliver PR routinely?<br>- Infrastructure/resources<br>- Motivation/willingness<br>- Patient demand/need<br>- Likelihood of profit/incentives<br>- Fit with existing practices<br>- Availability of trained staff<br>- Referral pathways (or lack of)

	Who are the key individuals, organisations, or decision-makers that can influence the routine delivery of PR services at the local level?<br><br>What national/local policy or guidelines may help with routine delivery of PR services in <country>?<br>Ask if they have heard about the lung health initiatives in <country>.<br>(e.g. Malaysian lung health initiative, any other initiatives specific to India/Bangladesh).
Sustainability	How could a sustainable PR service be implemented and rolled out in <country>?<br>What may be the policy, funding, resource requirements?<br>What could be the barriers and facilitators to sustained service delivery?
Summary	
Overall benefits	Perception of overall benefits (or not);<br>• to patients health and well-being<br>• to healthcare service in <country>
Any other comments?	
